# Adaptation and implementation of an employee mental health disclosure decision aid tool in a real-world sample

**DOI:** 10.1093/tbm/ibad072

**Published:** 2023-11-07

**Authors:** Elizabeth Stratton, Nick Glozier

**Affiliations:** Central Clinical School, Faculty of Medicine and Health University of Sydney, Sydney, Australia; ARC Centre of Excellence for Children and Families over the Life Course, Australia; Central Clinical School, Faculty of Medicine and Health University of Sydney, Sydney, Australia; ARC Centre of Excellence for Children and Families over the Life Course, Australia

**Keywords:** mental health, disclosure, workplace, decision aid tool

## Abstract

Making decisions about disclosing mental health conditions in the workplace is complicated. A previous randomized controlled trial showed that web-based decision aid tool (READY?) helped employees make decisions and improved mental health. We aimed to evaluate the implementation of this tool and its outcomes when scaled up by a governmental health and safety agency. We used website analytics and event data of those using the decision aid tool, and self-report stage of decision-making, distress, engagement, and usability data from consenting users of READY? over the first year of it being made publicly available. Over the year 2021, 100 adults opted in to be involved in the research evaluation of the program. This study replicated the previous Randomised Controlled Trial (RCT) that showed at post-intervention; a later stage of decision-making (*t*_1,99_ = 6.308, *P* < .001) with a large effect size (*d* = 0.87), and psychological distress was significantly reduced (*t*_1,99_ = 3.088, *P* < .001) with a moderate effect size (*d* = 0.41). READY? facilitated disclosure with 36.3% deciding to disclose after use. Disclosure was associated with a greater reduction in mental ill-health symptoms than non-disclosure [*F*(2,31) = 18.67, *P* < .001] with a moderate effect size (*d* = 0.64). Engagement, usage, and attrition rates were favourable when compared with other digital mental health approaches in community samples. This study shows that READY? is successfully implemented in a real-world sample. Aligning with the RCT results, for many, disclosure can be positive, research should continue to focus on developing organization-wide tools to create better supported and safe workplaces that promote disclosure.

## Teaser

Promoting workplace disclosure positively impacts mental health. In a real-world study, READY? an online disclosure decision aid tool, facilitated decision-making, and improved mental well-being.

**Implications**

**Research:** Future research should focus on developing and testing organization-wide tools outside of RCTs that foster supportive and safe workplaces, promoting mental health disclosure.

**Practice**: Practitioners can utilize the READY? decision aid tool to facilitate informed decision-making and reduce psychological distress when employees consider sharing their mental health conditions.

**Policy**: Policymakers should consider integrating tools like READY? into workplace health initiatives, supporting a culture of openness and communication around mental health, leading to improved employee well-being and productivity.

## Introduction

At a structural level, disclosure of a mental health condition is a necessary first step to help-seeking in the workplace, as employers are not legally obliged to provide individual support before disclosure. However the majority of employees with a mental health condition favour non-disclosure as their preferred option [1], and often disclose only when they are too unwell to continue working [2].

The primary reason for non-disclosure is fear of stigmatization and discrimination [3]. When questioned, employees are much less likely to report actual experiences of discrimination compared with their fear of it, possibly reflecting self-stigma [4]. Non-disclosure may protect individuals from the negative consequences associated with stigma and/or discrimination [5].

Decision-making around disclosure of a mental health condition in the workplace is often complex and highly individual. During the process of considering disclosure, individuals gather information and weigh up the positives and negatives. Decision aid tools are designed to help individuals make considered and deliberate choices. In the mental health context, we developed a web-based decision aid tool (READY?) to facilitate mental ill-health disclosure decisions for employees [6]. The original content of READY? used in the RCT was developed in a co-design approach fashion based upon currently available disclosure materials [7], guided by an international expert group. The content of READY? was co-developed in focus groups with employees who had disclosed mental ill-health in the workplace and key occupational decision-makers [3, 8] and further tested in an iterative fashion. The wording was specifically designed to be understandable by those with low literacy and has a Flesch-Kincaid Grade level of 6.6; i.e. understandable by the average 11 year old [9].

The final program (READY?) was based around a motivational interviewing approach and consisted of seven self-guided modules which enabled the user to consider potential consequences of (non)disclosure, Module 1: weighing advantages and obstacles, Module 2: needs for disclosure, i.e. workplace accommodations, Module 3: values to consider when disclosing, i.e. being open and honest, Module 4: when is the best time to tell, Module 5; reflecting on past disclosures, Module 6: who to tell, and Module 7: providing the user with an interactive summary their responses. The tool was designed to take ~5–10 minutes in total to complete. The program was carefully worded to avoid promoting any specific decision as the “correct” one.

Results from the RCT involving 107 employees [6], showed that READY? reduced decisional conflict, advanced decision-making stages, and led to a greater reduction in depressive symptoms. Notably, 28% of users chose to disclose and reported improved mental health. The READY? tool holds promise for wider implementation in diverse workplace settings in the real world but remains untested outside of trials settings. Hence this study of the adaptation in the real world.

### Is RCT evidence enough?

RCTs are the gold standard, and should remain so, when evaluating new interventions effectiveness. While digital approaches have shown efficacy in RCTs addressing mental health concerns in employees [10, 11], there is limited evidence of their effectiveness in “real-world” settings after the RCT phase. For instance, it is estimated that as few as 2% of smartphone applications for depression have reasonable evidence base outside of RCTs [12]. However, RCT results alone do not ensure the intervention can have meaningful impact on the wider population [13]. Three potential issues arise when scaling up RCTs into real-world populations. First, in RCTs, people meet pre-defined eligibility criteria and consent to take part in the trial, which makes them a highly selected sample and potential selection bias. Although this bias may be less pronounced in digital interventions [14].

The second issue, particularly pertinent to digital interventions in non-trial contexts is low uptake, as seen in one example of the MindSpot intervention. A free online supported mental health assessment and treatment website (https://www.mindspot.org.au/). Over 6 years, uptake of the online guided mental health treatment program was low with 18.7% of assessed users enrolled, lower than most RCTs [15].

Third, RCTs should be viewed as the start of the journey not the end point. True translation into practice occurs when researchers build collaboration with key stakeholders throughout the design and implementation [16] to deliver and evaluate the long-term use of interventions in a real-world setting.

This study evaluates the effectiveness and integration of READY? in this real-world setting.

## Methods

### Setting and recruitment

In an Australian context, a “Government health and safety agency” refers to a government organization or authority responsible for overseeing and regulating health and safety matters in the workplace. In Australia, workplace health and safety are critical areas of concern, and there are specific government agencies at the federal and state/territory levels that are dedicated to ensuring the safety and well-being of workers. These agencies play a crucial role in setting standards, providing resources, and enforcing regulations to protect workers’ health and safety in various industries and workplaces throughout Australia. The Recovery @ Work Toolkit, hosted by the State Insurance Regulatory Authority (SIRA), is a state initiative that offers evidence-based online resources to support mentally healthy workplaces for employers and employees. As part of this initiative, the READY? tool was implemented and made accessible to individuals who visited the Recovery @ Work website. The recruitment period for participants extended over 1 year, from August 2021 to September 2022. The target population consisted of employed working-age adults with a mental health condition who were considering their options regarding disclosure in the workplace.

Recruitment, data collection, and all aspects of the study were conducted entirely online. Detailed information about the READY? observational data collection was provided in the Participant Information Statement, which was provided upfront upon entry of the website and available for download. The study received ethical approval with the reference number (XXX). To participate, potential participants were required to complete an online form integrated into the READY? tool, signifying their consent to take part in the study.

### Adaptation

As it is a rarity for digital mental health digital to make it past RCT phase [12], we undertook extra measures necessary to ensure maximum uptake and engagement are reached before implementing READY? into the real world. We conducted three participatory focus groups via Zoom with 16 of SIRA’s lived experience reference group. Using aspects of our previous framework [8] where users were provided with the online version of READY? that was used in the RCT and asked their opinions on the language, content, and look and feel of the tool. The focus groups were not recorded, minutes were taken and required adaptations were agreed upon by all parties involved in the focus groups.

### Outcome measures embedded in the tool

Gender, age, and workplace relationships: (i) do you have a good relationship with your boss; (ii) do you have a good relationship with your co-workers? (both yes, no response).

### Primary

#### Stage of decision-making

This is a 5-question scale with a multiple choice selection for pre- and post-tool use, measures individual readiness to engage in decision-making [17]. Participants selected their stage from “I have not yet thought about the options” = 1 to “I have already told my employer” = 5.

#### Psychological distress

The Kessler Psychological Distress Scale (K10) is 10-item questionnaire intended to yield a 10–50 measure of distress based on questions about anxiety and depressive symptoms. A score <20 indicates participants are well, score from 20 to 24 indicates mild mental disorder, 25–29 indicates moderate mental disorder and >30 indicates severe mental disorder [18, 19]. The K10 had good internal consistency, with a Cronbach’s alpha of 0.83.

#### Usefulness

Three final questions were assessed after use (i) did you find this tool useful; (ii) would you recommend this tool to a co-worker; and (iii) any other feedback.

*Usage and Attrition* were automatically collected by the website and defined as the number of modules completed.

### Statistical analysis

All data were analysed using RStudio. Primary and secondary analyses were undertaken on an intention-to-treat (ITT) and per protocol (completers) basis with all consenting users. To ensure that an ITT approach is appropriate we examined the baseline differences between completers vs non-completers by using *t*-tests for continuous and chi-squared for binary measures. Only per protocol analysis was undertaken on one secondary analysis—comparing those who did and did not choose to disclose.

MICE (Multivariate Imputation via Chained Equations) was used to impute missing data for the ITT analysis by creating multiple imputations as compared with a single imputation (such as mean) takes care of uncertainty in missing values. MICE assumes that the missing data are missing at random, which means that the probability that a value is missing depends only on observed value and can be predicted using them. It imputes data on a variable-by-variable basis by specifying an imputation model per variable and is considered a conservative approach [13]. Sensitivity analysis was used to compare ITT and completers change scores.

Effectiveness was analysed by calculating changes between baseline and post-intervention scores using paired samples *t*-tests for key decision and mental health variables. Cohen’s *d* was calculated by comparing the baseline scores to the post-intervention difference in mean. According to Cohen, *d* = 0.2 can be considered small, *d* = 0.5 a moderate effect, and *d* = 0.8 a large effect [20]. Independent sample *t*-tests or Pearson’s *r* were used to evaluate whether there were differences in change scores between those completers who chose to disclose or not. Analysis of covariance was used to explore change scores while adjusting for baseline scores.

Usage, module use, and disclosure rates are presented using means and standard deviation (SD) or prevalence rates where appropriate.

## Results

Several adaptions were made after the focus groups regarding the language, look and feel, and content of READY?.

### Language

Overall, participants in the focus groups expressed a preference not to use the term “mental health conditions.” To address this, we decided to simply use the term “mental health” throughout the tool. The phrase “tell” was considered inappropriate and the words “share” or “talk” were used when referring to disclosing.

### Imagery

The original RCT version of READY? had images behind the text on each landing page. A majority of the focus group members advised this made the text difficult to read. We decided to remove the images from behind the text and have a plain background.

From August 2021 to August 2022, *n* = 100 users opted into the research and became study participants, of whom *n* = 33 (33%) provided follow-up data. While it might be deemed low within a clinical trial, it’s crucial to comprehend real-world adoption in a naturalistic sample. Thus, this follow-up rate should not equate to a clinical trial setting.

The average age was 41.02 (SD = 10.17) years. Predominately female *n* = 72 (72%). Majority reported having a good relationship with their boss *n* = 71 (71%) and colleagues *n* = 81 (81%). At Baseline on average participants were at a level of “I am considering my options” on the stage of decision-making scale and reported a moderate level of distress [19] (Table 1).

**Table 1:** Baseline characteristics of READY? participants comparing study completer vs non-completers

Variable	Overall (*n* = 100)	Completers (*n* = 33)	Non-completers (*n* = 67)	significance
	*N* (%)	*N* (%)	*N* (%)	Chi-squared
Sex (female)	72 (72.00)	20 (60.61)	52 (77.61)	*X* ^2^ = 3.287, *P* = .070
Good relationship boss (yes)	71 (71.00)	23 (69.70)	48 (71.64)	*X* ^2^ = 0.100, *P* = .751
Good relationship colleagues (yes)	81 (81.00)	23 (69.70)	58 (86.57)	*X* ^2^ = 4.542, *P* = .033

	*M* (SD)	*M* (SD)	*M* (SD)	*t*-test
Age	41.02 (10.17)	42.52 (10.03)	40.27 (10.23)	*t* = 1.034, *P* = .304
Stage of decision-making (SDM)	3.53 (1.24)	3.36 (1.31)	3.61 (1.21)	*t* = 0.939, *P* = .350
Psychological distress (K10)	28.09 (9.12)	27.36 (8.69)	28.45 (9.37)	*t* = 0.557, *P* = .579

All participants (*n* = 100) started the intervention, Module 1 was completed by 68 of the participants (68%), Modules 2 and 3 by *n* = 51 (51%), Modules 4–7 by *n* = 36 (36%). On average users completed 2.8 (SD = 2.1) modules.

Usefulness. Almost all respondents found READY? useful (*n* = 25, 75.76%), or possibly useful (*n* = 7, 21.21%). Similarly, *n* = 24 (72.73%) participants stated they would recommend READY? to a colleague, *n* = 8 (24.24%).

Only one baseline item differed between participants who persisted with the study (study completers) and those lost to follow-up; non-completers reported better relationships with their colleagues (Table 1).

**Figure 1:**
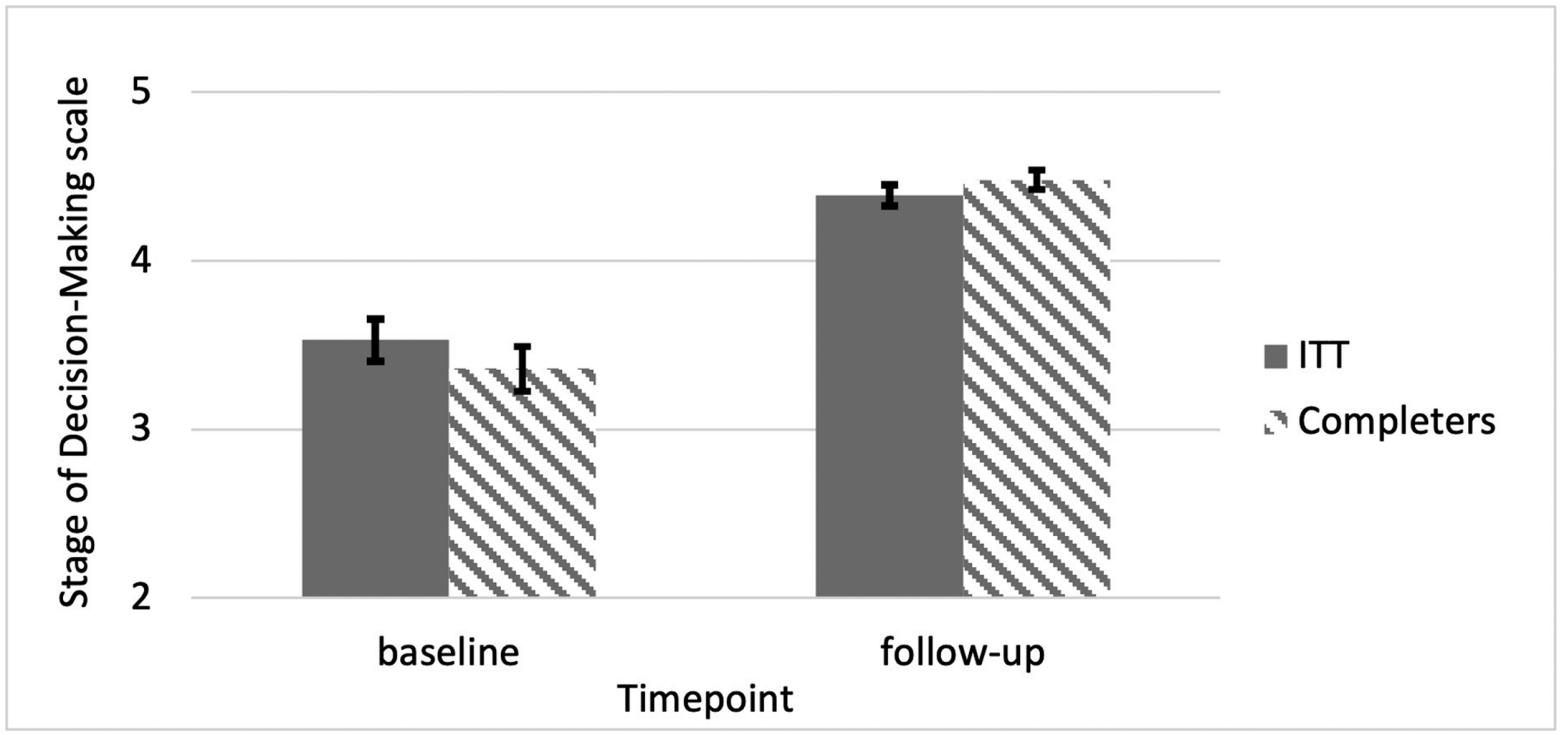
Changes in stage of decision-making mean scores and standard errors over the observation period.

At post-intervention, participants were at a later stage of decision-making moving on average to “I am close to making a decision” utilizing a conservative ITT approach (*t*_1,99_ = 6.308, *P* < .001) with a large effect size of (*d* = 0.87) (Fig. 1). Similar results were observed in those that completed the follow-up (*t*_1,32_ = 4.501, *P* < .001) (Fig. 1). Sensitivity analysis shows there was no significant difference in change scores between ITT and completers (*t*_2,98_ = 1.350, *P* = .180).

**Figure 2:**
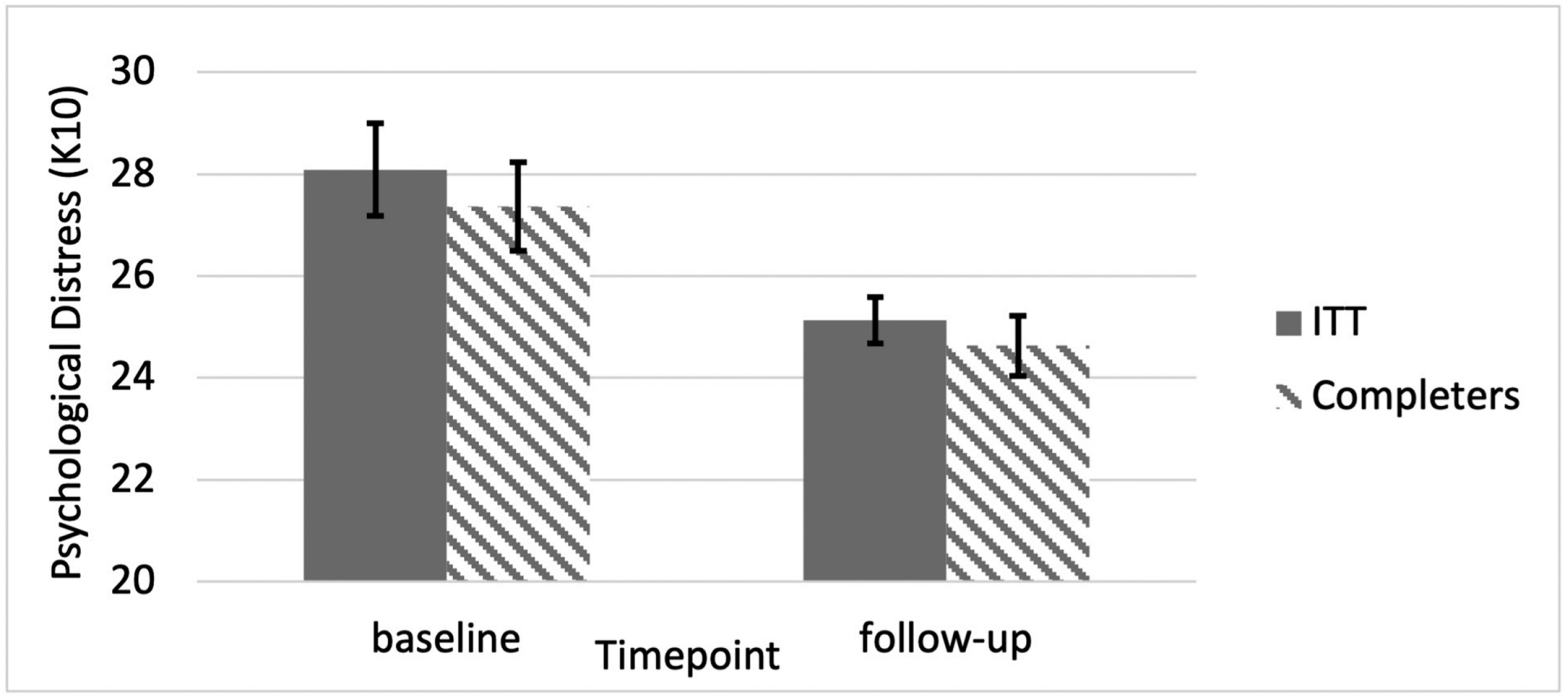
Changes in psychological distress scores estimated marginal mean scores and standard errors over the observation period.

Psychological distress was significantly reduced at post-intervention in the ITT sample (*t*_1,99_ = 3.088, *P* < .001) with a moderate size of (*d* = 0.41). In the overall ITT analysis participants on average moved from the upper end of moderate levels of distress (*M* = 28.09, SD = 9.12), to lower end of the scale of moderate symptoms of distress (*M* = 25.13, SD = 4.51). A score from 25 to 29 indicates a moderate mental disorder [19] (Fig. 2). Replicating the reduction in mental ill-health symptoms observed in the RCT [6]. Sensitivity analysis shows there was no significant difference in change scores between ITT and completers (*t*_2,98_ = 0.037, *P* = .970).

**Figure 3:**
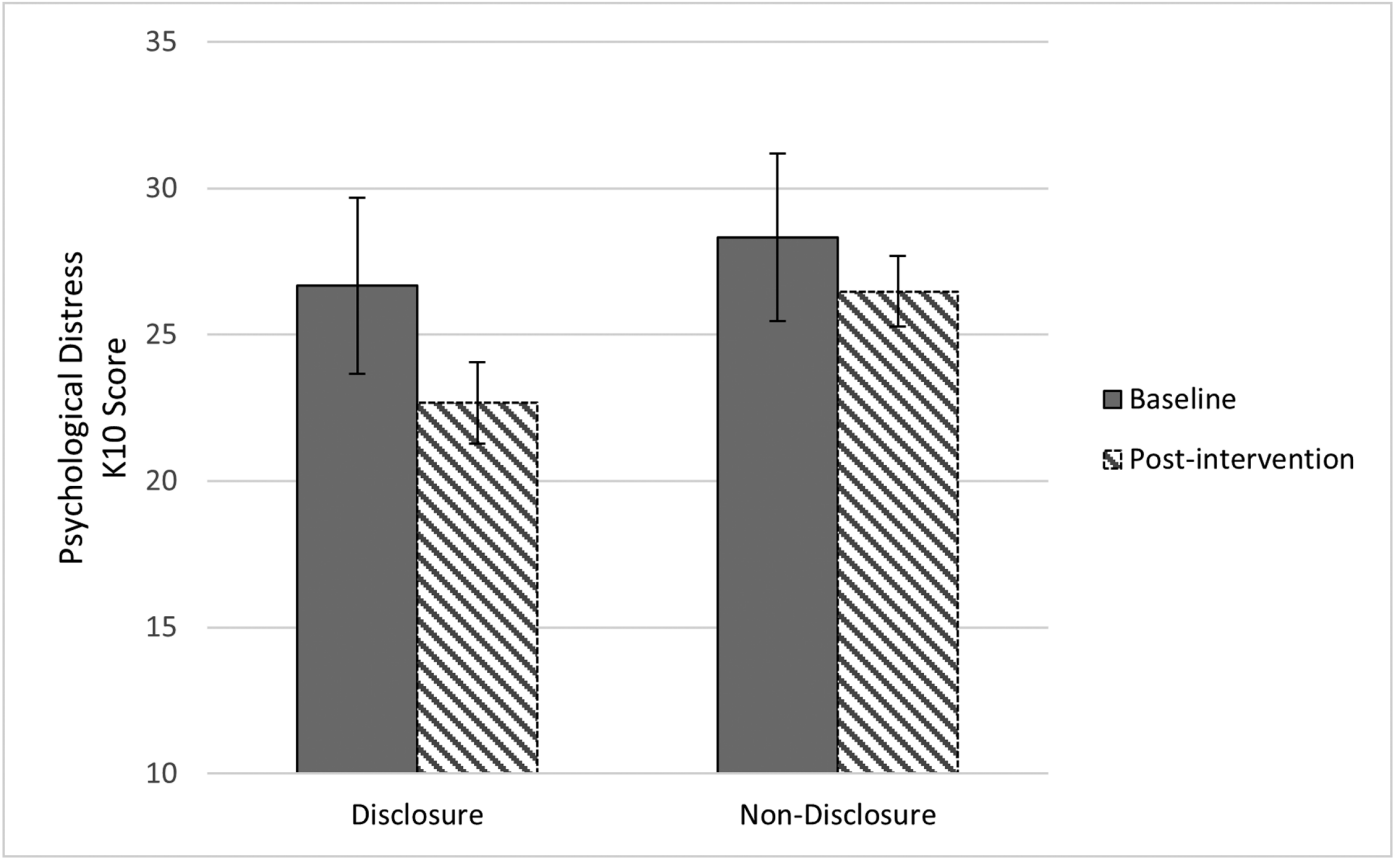
Mean change scores psychological distress disclosure (*n* = 12) vs non-disclosure (*n* = 21).

Similar results were observed in those that completed the follow-up (*t*_1,32_ = 2.620, *P* = .007) (Fig. 3).

Of the 33 participants that completed the post-intervention questions, 13 (39.4%) had made a disclosure decision, of whom 12 decided to disclose, and one decided not to disclose. Those not deciding were assumed to have not disclosed. There were no significant differences at baseline in those that disclosed compared with those that did not disclose (Table 2).

**Table 2:** Baseline differences in disclosure vs non-disclosure groups

Variable	Disclosure (*n* = 12)	Non-disclosure (*n* = 21)	Significance
	*N* (%)	*N* (%)	Chi-squared
Sex (female)	6 (50.00)	14 (66.70)	*X* ^2^ = 0.888, *P* = .346
Good relationship boss (yes)	9 (75.00)	14 (66.70)	*X* ^2^ = 0.683, *P* = 0.711
Good relationship colleagues (yes)	9 (75.00)	17 (81.00)	*X* ^2^ = 1.088, *P* = .580

	*M* (SD)	*M* (SD)	*t*-test
Age	44.67 (9.89)	41.29 (10.14)	*t* = 0.929, *P* = .360
Stage of decision-making (SDM)	3.48 (1.21)	3.17 (1.53)	*t* = 0.643, *P* = .525
Psychological distress (K10)	26.67 (9.02)	28.33 (8.56)	*t* = 0.844, *P* = .405

Disclosure was associated with a greater reduction in mental ill-health symptoms (mean difference = −4.00) than non-disclosure (mean difference = −1.23), [*F*(2,31) = 18.67, *P* < .001] with moderate effect size (*d* = 0.64) (Fig. 3). The group who disclosed improved clinically from moderate to mild psychological distress.

## Discussion

This study presents the results of the implementation of a mental ill-health disclosure decision aid tool in a real-world setting. We replicated the findings from the earlier RCT [6] that the use of READY? enabled participants to move to later stage of decision-making and was associated with a reduction in psychological distress. Most of the participants who completed READY? found it useful and would recommend to a colleague.

This represents the only real-world study of an online mental health disclosure decision aid tool, bringing greater insight into use and effectiveness outside the constraints of a research trial. However, there are limitations. There were 768 unique users who visited the website over a 1-year period of whom fewer than one in seven provided informed consent (the *n* = 100 included in this study) to take part in the evaluation. As such we do not know whether these results generalize to the other users. Since only a fraction of the users provided informed consent, it would be helpful to know more about the characteristics of those who consented and how they compared with the larger user population. This would provide additional insights into the generalizability of the findings and should be consideration for the future use of this, or similar tools.

There was a 66% non-completion rate, potentially causing attrition bias, although this seemed unlikely from the baseline comparison, and partially addressed by the analytic method. Furthermore, retention rates in this sample (33%) are significantly improved compared with other naturalistic comparisons where as little as 0.5% response rate to non-compulsory data is reported [21]. We hypothesis that this is because (i) users are a specific group who are motivated to seek and answer for their questions, (ii) the intervention is designed for one-time use and is much shorter than other interventions, (iii) our study collected follow-up data soon after completion which may have resulted in an increased rate of retention. However, we acknowledge that this likely has substantial implications for the implementation of this intervention in this setting.

Furthermore, logistically, we only collected feedback on those that completed the intervention, as a post-intervention survey, this may not reflect all varied user experiences, such as drop out because usefulness/unusefulness. Understanding why some dropped out could influence the tool’s acceptability and satisfaction. This insight is crucial for enhancing its future utility for individuals facing mental health disclosure decisions in the workplace.

In this real-world setting 39% decided to disclose after using READY?, similar to the rate of disclosure observed in the RCT [6]. Although the optimum rate of disclosure is yet to be established, we now have two studies suggesting that disclosure rates may be approximately one-third.

The facilitation of disclosure is particularly important as there is increasing evidence to suggest that disclosing may be a good thing. Being open about mental health conditions reduces self-stigmatization and increases a sense of power and control [22, 23]. Those who used READY? both in the RCT [6] and in this study experienced a reduction in mood and stress symptoms. The effect was smaller in those who decided not to disclose, confirming that disclosure appears to be good for employee’s mental health. Despite these findings, employees are often faced with stories of negative experiences, and attitudes towards disclosure [24], leaving them reluctant to disclose [23]. Our findings highlighting the importance of addressing these negative scenarios in an individualized way by enabling people to consider potential benefits of disclosure. Organizations can play a role in changing the conversation by flipping the narrative to promoting what employees will have access to, and how they can thrive with support.

In the RCT [6] 46% of participants completed all of the modules, whereas 36% completed all modules in this study. Mean module completion also differed with the real-world group reporting fewer average modules completed (2.8) compared with the RCT (3.9 modules). A potential reason for this is that the RCT group are a highly selected and likely more motivated sample, creating bias.

Interestingly, usage and engagement (for those who provided consent) reported here in a real-world setting is higher than found in systematic review evidence of the uptake of digital mental health approaches in general community settings which suggest that 0.5%–28% complete all modules [25] compared with the 36% observed in this study. This is likely due to four factors. First, motivation. Users of READY? are looking for information provided on disclosure decision-making. READY? is tailored to assist users in making a specific deliberate choice and likely increases motivation to continue use. Motivation is increased in interventions that consider readiness to change and motivational techniques [26] which is the foundation that READY? is built on.

Second, sustainability. A key aspect of interventions delivered in the real world are their ability to be implemented on an ongoing basis in the community by existing providers in a financially feasible way [26]. Ongoing collaboration and development with the community and industry groups is critical to ongoing success, particularly with recruitment and advertisement to community groups. Researchers should consider maintaining relationships as a necessity for ongoing successful sustainability and engagement post-RCTs.

Third, the online tool allows for the freedom and autonomy to access disclosure information anywhere, at any time. Under a shared theoretical framework [27] of motivational interviewing [28] and self-determination theory [29], autonomy is a particularly important part in decision-making, as a component of self-determination, or the ability to make one’s own decisions. Self-determination has been linked to increased self-confidence, the more control individuals have over the decisions in their life the more confident they feel [30]. READY? allows employees to make a well-informed decision by autonomous disentangling disclosure decisions. When developing interventions in this space, developers and researchers alike should consider the use of “client-centred” autonomy to promote the desired change outcome.

Finally, READY? was co-designed with end-users for the RCT and when implementing in the real-world setting. This approach doubtlessly resulted in the development of an effective and engaging tool. Using end-user approaches to consider the barriers, facilitators, design, and implementation ensured that the tool was effective and useful to the intended target audience [16]. Future designs should consider these two design aspects as key to ensure optimum usability, engagement, and benefit.

In conclusion, this study offers valuable insights into the real-world implementation of a mental health disclosure decision aid tool. Replicating earlier findings, the READY? tool facilitated more advanced decision-making stages and reduced psychological distress among users. The study’s significance lies in being the only real-world investigation of an online mental health disclosure tool, offering insights beyond controlled research settings. While acknowledging limitations such as the low consent rate and potential bias, the study underscores the tool’s potential to promote disclosure and its positive impact on mental health. The higher engagement and usage rates observed compared with traditional digital mental health interventions can be attributed to user motivation, sustainability, autonomy, and user-centred design. The study encourages future research to focus on addressing barriers to disclosure, maintaining community relationships, promoting autonomy, and employing user-centred design to ensure the effectiveness and usability of interventions in this crucial area.

## Transparency Statements

(1) *Study registration*. Study registration involves declaring the study design, variables, and treatment conditions in an independent, institutional repository(e.g. http://clinicaltrials.gov, http://openscienceframework.org, http://socialscienceregistry.org/). A study is considered pre-registered if it is registered in a repository prior to the study began. Consider the following examples: this study was not formally registered. (2) *Analytic plan pre-registration*. Pre-registration of an analytic plan involves specification of the planned sequence of analyses or the statistical model that will be reported. Reporting the planned outcome variables alone does not qualify as analytic plan pre-registration. Consider the following examples: the analysis plan was not formally pre-registered. (3) *Data availability.* Indicate whether the data underlying the presented analyses will be made available in a third-party archive/repository. If data is available in an archive, indicate if this is fully available to the public or has protected access (available for limited audience, with clearly defined requirements and steps to obtain data). If data is not in a public archive, describe any steps needed to access the data. Consider the following examples: de-identified data from this study are not available in a public archive. De-identified data from this study will be made available (as allowable according to institutional IRB standards) by emailing the corresponding author. (4) *Analytic code availability.* Analytic code refers to the programming code used to conduct the analyses in the statistical software. Consider the following examples: analytic code used to conduct the analyses presented in this study are not available in a public archive. They may be available by emailing the corresponding author. (5) *Materials availability.* Materials include any stimuli used in this study, intervention protocols, and survey instruments and items. Consider the following examples: materials used to conduct the study are not publically available.

## References

[1] Lasalvia A , Zoppei S, Van Bortel T, et al.; ASPEN/INDIGO Study Group. Global pattern of experienced and anticipated discrimination reported by people with major depressive disorder: a cross-sectional survey. Lancet 2013;381:55–62. 10.1016/S0140-6736(12)61379-8 23083627

[2] Toth KE , Dewa CS. Employee decision-making about disclosure of a mental disorder at work. J Occup Rehabil 2014;24:732–46. 10.1007/s10926-014-9504-y 24604575

[3] Stratton E , Einboden R, Ryan R, et al. Deciding to disclose a mental health condition in male dominated workplaces; a focus-group study. Front Psychiatry 2018;9:684. 10.3389/fpsyt.2018.00684 30618865 PMC6305748

[4] Waugh W , Lethem C, Sherring S, et al. Exploring experiences of and attitudes towards mental illness and disclosure amongst health care professionals: a qualitative study. J Ment Health 2017;26:457–63. 10.1080/09638237.2017.1322184 28488890

[5] MacDonald-Wilson KL , Russinova Z, Rogers ES, et al. Disclosure of mental health disabilities in the workplace. In: Schultz IZ, Rogers ES (eds.), Work Accommodation and Retention in Mental Health. New York, NY: Springer New York, 2011, 191–217.

[6] Stratton E , Choi I, Calvo R, et al. Web-based decision aid tool for disclosure of a mental health condition in the workplace: a randomised controlled trial. Occup Environ Med 2019;76:595–602. 10.1136/oemed-2019-105726 31413183

[7] Henderson C , Brohan E, Clement S, et al. Decision aid on disclosure of mental health status to an employer: feasibility and outcomes of a randomised controlled trial. Br J Psychiatry 2013;203:350–7. 10.1192/bjp.bp.113.128470 24072755

[8] Stratton E , Choi I, Peters D, et al. Co-designing a web-based decision aid tool for employees disclosure of mental health conditions: a participatory study design using employee and organizational preferences. JMIR Form Res 2020;4:e23337. 10.2196/23337 33155982 PMC7679208

[9] Kincaid PJ , Fishburne, RP, Rogers, RL, et al. “Derivation of New Readability Formulas (Automated Readability Index, Fog Count and Flesch Reading Ease Formula) for Navy Enlisted Personnel” Memphis, Tenn. Naval Air Station: Institute for Simulation and Training, 1975.

[10] Stratton E , Lampit A, Choi I, et al. Effectiveness of eHealth interventions for reducing mental health conditions in employees: a systematic review and meta-analysis. PLoS One 2017;12:e0189904. 10.1371/journal.pone.0189904 29267334 PMC5739441

[11] Stratton E , Lampit A, Choi I, et al. Trends in effectiveness of organizational eHealth interventions in addressing employee mental health: systematic review and meta-analysis. J Med Internet Res 2022;24:e37776. 10.2196/37776 36166285 PMC9555335

[12] Anthes E. Mental health: there’s an app for that. Nature 2016;532:20–3. 10.1038/532020a 27078548

[13] van Buuren S , Groothuis-Oudshoorn K. mice: multivariate imputation by chained equations in R. J Stat Softw 2011;45:1–67.

[14] Donkin L , Hickie IB, Christensen H, et al. Sampling bias in an internet treatment trial for depression. Transl Psychiatry 2012;2:e174. 10.1038/tp.2012.100 23092978 PMC3565809

[15] Titov N , Dear BF, Nielssen O, et al. User characteristics and outcomes from a national digital mental health service: an observational study of registrants of the Australian MindSpot Clinic. Lancet Digit Health 2020;2:e582–93. 10.1016/S2589-7500(20)30224-7 33103097 PMC7571905

[16] Bear HA , Ayala Nunes L, DeJesus J, et al. Determination of markers of successful implementation of mental health apps for young people: systematic review. J Med Internet Res 2022;24:e40347. 10.2196/40347 36350704 PMC9685513

[17] O’Connor AM , Stacey D, Rovner D, et al. Decision aids for people facing health treatment or screening decisions. Cochrane Database Syst Rev 2001;3:CD001431. 10.1002/14651858.CD001431 11686990

[18] Kessler RC , Barker PR, Colpe LJ, et al. Screening for serious mental illness in the general population. Arch Gen Psychiatry 2003;60:184–9. 10.1001/archpsyc.60.2.184 12578436

[19] Andrews G , Slade T. Interpreting scores on the Kessler Psychological Distress Scale (K10). Aust N Z J Public Health 2001;25:494–7. 10.1111/j.1467-842x.2001.tb00310.x 11824981

[20] Cohen J. Statistical Power Analysis for Behavioural Sciences. Hillsdale, NJ: Lawrence Erlbaum Associates, 1988.

[21] Christensen H , Griffiths KM, Jorm AF. Delivering interventions for depression by using the internet: randomised controlled trial. BMJ 2004;328:265. 10.1136/bmj.37945.566632.EE 14742346 PMC324455

[22] Corrigan PW , Morris S, Larson J, et al. SELF-STIGMA AND COMING OUT ABOUT ONE’S MENTAL ILLNESS. J Commun Psychol 2010;38:259–75. 10.1002/jcop.20363 PMC3747968 23970807

[23] Corrigan PW , Rao D. On the self-stigma of mental illness: stages, disclosure, and strategies for change. Can J Psychiatry 2012;57:464–9. 10.1177/070674371205700804 22854028 PMC3610943

[24] Reavley NJ , Jorm AF, Morgan AJ. Discrimination and positive treatment toward people with mental health problems in workplace and education settings: findings from an Australian National Survey. Stig Health 2017;2:254–65. 10.1037/sah0000059

[25] Fleming T , Bavin L, Lucassen M, et al. Beyond the trial: systematic review of real-world uptake and engagement with digital self-help interventions for depression, low mood, or anxiety. J Med Internet Res 2018;20:e199. 10.2196/jmir.9275 29875089 PMC6010835

[26] Marchand E , Stice E, Rohde P, et al. Moving from efficacy to effectiveness trials in prevention research. Behav Res Ther 2011;49:32–41. 10.1016/j.brat.2010.10.008 21092935 PMC3883560

[27] Vansteenkiste M , Williams GC, Resnicow K. Toward systematic integration between self-determination theory and motivational interviewing as examples of top-down and bottom-up intervention development: autonomy or volition as a fundamental theoretical principle. Int J Behav Nutr Phys Act 2012;9:23. 10.1186/1479-5868-9-23 22385828 PMC3315422

[28] Miller WR , Rose GS. Toward a theory of motivational interviewing. Am Psychol 2009;64:527–37. 10.1037/a0016830 19739882 PMC2759607

[29] Deci EL , Ryan RM. The “what” and “why” of goal pursuits: human needs and the self-determination of behavior. Psychol Inq 2000;11:227–68. 10.1207/s15327965pli1104_01

[30] Piltch CA. The role of self-determination in mental health recovery. Psychiatr Rehabil J 2016;39:77–80. 10.1037/prj0000176 26974741

